# The Properties of Thin Films Based on Chitosan/Konjac Glucomannan Blends

**DOI:** 10.3390/polym16213072

**Published:** 2024-10-31

**Authors:** Karolina Kulka-Kamińska, Alina Sionkowska

**Affiliations:** Department of Biomaterials and Cosmetic Chemistry, Faculty of Chemistry, Nicolaus Copernicus University, Gagarin 7 Street, 87-100 Torun, Poland

**Keywords:** chitosan, konjac glucomannan, blends, miscibility, biopolymer film

## Abstract

In this work, blend films were prepared by blending 2% chitosan (CS) and 0.5% konjac glucomannan (KGM) solutions. Five ratios of the blend mixture were implemented (95:5, 80:20, 50:50, 20:80, and 5:95), and a pure CS film and a pure KGM film were also obtained. All the polymeric films were evaluated using FTIR spectroscopy, mechanical testing, SEM and AFM imaging, thermogravimetric analyses, swelling and degradation analyses, and contact angle measurements. The CS/KGM blends were assessed for their miscibility. Additionally, the blend films’ properties were evaluated after six months of storage. The proposed blends had good miscibility in a full range of composition proportions. The blend samples, compared to the pure CS film, indicated better structural integrity. The surface structure of the blend films was rather uniform and smooth. The sample CS/KGM 20:80 had the highest roughness value (Rq = 12.60 nm). The KGM addition increased the thermal stability of films. The blend sample CS/KGM 5:95 exhibited the greatest swelling ability, reaching a swelling degree of 946% in the first fifteen minutes of the analysis. Furthermore, the addition of KGM to CS improved the wettability of the film samples. As a result of their good mechanical properties, surface characteristics, and miscibility, the proposed CS/KGM blends are promising materials for topical biomedical and cosmetic applications.

## 1. Introduction

The modern industry focuses on ensuring sustainable and holistic development, with an emphasis on safe and environmentally friendly solutions. Biopolymers are a group of biomaterials with significant potential in these areas due to their biodegradability, biocompatibility, and non-toxicity [1,2]. Various modifications of biopolymers are used in the search for new, more efficient materials. A contemporary methodology in numerous disciplines is the creation of composite polymeric materials. These materials are characterized by better physicochemical, mechanical, and stability properties compared to their constituent starting polymers [3].

Two principal techniques for obtaining blends are identified by the extant literature: the first involves mixing in a molten state, while the second utilizes polymeric solutions combined in a suitable solvent [3,4]. Nevertheless, the second method appears to be more pertinent for biopolymers, as a means of preventing the degradation of these natural components when subjected to elevated pressure and temperature during the process of melt mixing [3]. Blend composites are frequently used in the form of film, hydrogel, sponge, or fiber and can be applied as a wound dressing, scaffold, membrane, or even a drug-encapsulating agent [5,6,7,8]. An essential quality of blends is their miscibility, which can be evaluated through a few straightforward studies. These include the assessment of the optical homogeneity of the mixture, the determination of the glass transition temperature, and the analysis of molecular-level interactions [3].

The biopolymer that has gained the most popularity over the past two decades, primarily due to its advantageous characteristics, is chitosan, a chitin derivative. The biomacromolecule is composed of 2-acetamido-2-deoxy-D-glucopyranose and 2-amino-2-deoxy-D-glucopyranose units [9]. In addition to exhibiting biodegradability, non-toxicity, and biocompatibility, the biopolymer displays adsorption properties and demonstrates antioxidant and antimicrobial activity. These properties are primarily attributable to the biopolymer’s polycationic nature [10,11]. Chitosan is soluble in water only in an acidic environment, whereby the amino groups are protonated. This biomolecule can be derived from a variety of sources of chitin, including fungi, marine organisms, certain algae, insects, and microorganisms [12]. The preparation of chitin can be achieved through two principal methods: chemical and biological [13]. In the pursuit of materials with optimal properties for a given application, chitosan modifications are employed. The presence of functional groups at C-2 (-NH_2_), C-3 (secondary -OH), and C-6 (primary -OH) renders chitosan susceptible to chemical modifications [10]. Chitosan derivatives are produced through chemical reactions, while another approach is polymer cross-linking. As previously stated, additional methods of chitosan modification entail the formation of polymer blends [14]. Chitosan-based materials have a wide range of applications in medicine, pharmaceuticals, food, packaging, cosmetics industries, and water purification. This demonstrates the unique properties of chitosan and its high potential as a material.

Konjac glucomannan (KGM) is a neutral polysaccharide that is isolated from Amorphophallus konjac plant tubers. This biopolymer is composed of D-mannose and D-glucose units with a molar ratio of 1.6:1. The units are linked by β-(1,4) glycosidic bonds [15,16]. Additionally, a minor proportion of β-1,3-linkages are present at the mannose C-3 position—the konjac glucomannan chain contains a randomly distributed number of acetyl groups, which represent 5 to 10% [17]. The high affinity of the KGM for water is precisely the merit of the acetyl and hydroxyl groups’ presence [18]. KGM displays high viscosity, a robust water-holding capacity, and excellent gel-forming properties in aqueous solutions [19]. Furthermore, it functions as an effective emulsifier and demonstrates remarkable film-forming capabilities [20]. This polysaccharide is a valuable macromolecular raw material used in the food industry, as well as increasingly in the biomedical and cosmetic fields. Nevertheless, the utilization of KGM-based materials in numerous fields is frequently constrained by their inadequate mechanical properties, which can be attributed to the high hydroxyl group content in the polymeric backbone [21].

There are reports in the literature on chitosan/glucomannan blends, which are mainly used for drug-coating applications [22], wound dressing [18], peptide and protein delivery vehicles [23], and membranes [24]. These reports are supplemented by a general survey describing the proposed blend as a potential matrix biomaterial [25], as well as the influence of gamma irradiation on such blend materials as a part of the sterilization process [26].

In this study, we were focused on obtaining a new material based on biopolymers. This paper aimed to evaluate the properties of chitosan/konjac glucomannan blend films. For this purpose, infrared spectroscopy, a mechanical parameters evaluation, and a thermogravimetric analysis were carried out. Additionally, the surface properties and morphology of the materials were evaluated through contact angle measurements, scanning electron microscopy, and atomic force microscopy. The films were prepared from different blend proportions. The characteristics of the blends were compared to the pure chitosan and the pure glucomannan films. The same characterization tools were used for the films’ properties evaluation after a six-month period of storage.

## 2. Materials and Methods

### 2.1. Materials

Low-molecular-weight chitosan (CS) and konjac glucomannan (KGM) (M_vCS_ = 7.31 × 10^5^ g/mol; M_vKGM_ = 7.71 × 10^5^ g/mol) were purchased from the POL-AURA company (Dywity, Poland). Acetic acid was acquired from POCH (Gliwice, Poland). Phosphate Buffer Saline (PBS), in the form of tablets, was acquired from Life Technologies Limited (Renfrew, UK).

### 2.2. Chitosan/Konjac Glucomannan Films Preparation

**Figure 1:**
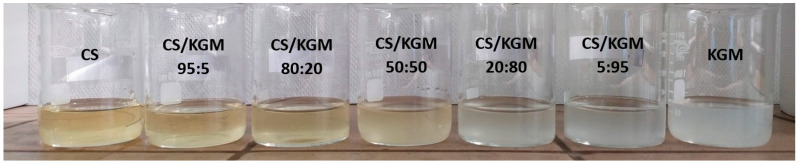
Overview of chitosan (CS), konjac glucomannan (KGM), and their blends’ solution appearance.

All of the films prepared during this research were prepared by the solvent casting method. Solutions of 2% (*w*/*v*) chitosan (CS) and 0.5% (*w*/*v*) konjac glucomannan (KGM) were prepared by dissolving the polymers in 0.1 M acetic acid. To prepare the control samples, 25 g of CS pure solution and 25 g of KGM pure solution were poured out separately on polystyrene plates (10 cm × 10 cm). Subsequently, the samples were placed in an incubator, which was set at 37 °C, and allowed to dry. To prepare the blend samples, the CS and the KGM solutions were mixed in weight ratios of 95:5, 80:20, 50:50, 20:80, and 5:95. The blended solutions were stirred for 24 h and then poured onto polystyrene plates. They were dried in the same manner as the control samples. The drying process took 3 to 5 days, depending on the sample. The pictures of prepared solutions and films are presented in Figure 1 and Table 1, respectively.

The films, which were examined after 6 months, were stored during this time period without access to light, at room temperature, at a humidity of approximately 50%.

### 2.3. Fourier Transform Infrared Spectroscopy (FTIR)

The FTIR spectra of all the prepared films were assessed using a Nicolet iS10 spectrophotometer equipped with a diamond attenuated total reflectance (ATR) unit (Thermo Fisher Scientific, Waltham, MA, USA). The samples were scanned in the wavenumber range of 4000–400 cm^−1^ with a 4 cm^−1^ resolution using 64 scans, in accordance with the methodology employed in previous research [27]. The measurements were repeated on the same samples after a period of 6 months. OMNIC (version 9.2.86) and Excel (version 2409) software were used to analyze and process data.

### 2.4. Mechanical Testing

The mechanical parameters were assessed under room conditions, using a mechanical testing machine (Z.05, Zwick and Roell, Ulm, Germany). The specimens were cut in the same way, maintaining a similar shape, with a constriction of approximately 4 mm [28]. The measurement parameters were as follows: the speed starting position was 50 mm/min, the speed of the initial force was 5 mm/min, and the initial force was 0.1 MPa. The evaluation included the assessment of the Young’s modulus, tensile strength, and elongation at the breaking point. The data were collected using the TestXpert II 2017 program and were subsequently presented as average values with standard deviations. A Q-Dixon test was performed to identify and reject outliers. A one-way ANOVA test was applied to determine statistically significant differences between samples; the reference samples were the initial polymers films. The mechanical properties of the 6-month-old samples were also evaluated and compared to the freshly obtained samples.

### 2.5. Scanning Electron Microscopy (SEM)

The morphology of the obtained films was studied using a scanning electron microscope, manufactured by LEO Electron Microscopy Ltd. (Model 1430 VP, Cambridge, UK,). The samples were covered with gold to provide a conductive surface for the electron beam interaction [29]. The magnification of all of the SEM images presented below is 10,000×.

### 2.6. Atomic Force Microscopy (AFM)

The surface of the polymer samples was analyzed using an atomic force microscope. The images were obtained by a MultiMode Scanning probe microscope, the NanoScope IIIa (Digital Instruments Veeco Metrology Group, Santa Barbara, CA, USA). The apparatus was operating in a tapping mode, at room temperature, in an air atmosphere [29]. The roughness parameters were calculated from 5.0 μm-by-5.0 μm scanned areas using Gwyddion software (version 2.62).

### 2.7. Thermogravimetric Analysis

The thermogravimetric assessment was conducted using an SDT 2960 Simultaneous TGA-DTA analyzer from TA Instruments (TA Instruments Manufactures, Eschborn, Germany). The analysis was carried out over a temperature range from 25 °C to 600 °C, at a heating rate of 20 °C/min, in a nitrogen atmosphere [29].

### 2.8. Swelling and Degradation Properties

The CS/KGM films were cut into squares of similar weights. The samples were dried for 24 h at 45 °C. Each type of sample (five squares in one series) was placed in a container with 50 mL Phosphate Buffer Saline (PBS) at 37 °C [29]. The measurements of the samples’ weights were conducted after 15 min, 1 h, 2 h, 4 h, 8 h, 24 h, 48 h, 72 h, 1 week, and 2 weeks. Following an appropriate interval, the samples were taken out from the PBS solution, and, subsequently, the excess fluid was removed using paper. Thereafter, the samples were weighed. The measurements were taken fresh from the collection. They were repeated on samples that were 6 months old. The swelling degrees were calculated using the following equation:

Swelling = (m_t_ − m_0_)/m_0_ × 100% [%] (1)

m_t_—the weight of the material after immersion in PBS [g];
m_0_—the initial weight of the material [g].

### 2.9. Contact Angle and Surface Free Energy

The contact angle measurements of the obtained films were used to calculate the surface free energy (γ_s_) and its polar (γ_sp_) and dispersive (γ_sd_) components, by the Owens–Wendt method [29]. The contact angle values of two liquids (glycerine (G) and diiodomethane (D)) were measured via a goniometer that was equipped with a system for drop shape analyses (DSA 10 produced by Krüss, Hamburg, Germany). The result of the contact angle for each sample is an average value with a standard deviation (SD). The Q-Dixon test was implemented to identify and reject outliers. All the measurements were carried out at a constant temperature value.

## 3. Results

### 3.1. Fourier Transform Infrared Spectroscopy (FTIR)

The chemical structure of initial polysaccharides was confirmed with the FTIR analysis. The initial infrared spectra of the pure chitosan and the pure konjac glucomannan films are characteristic of them and show several main bands.

In the konjac glucomannan IR spectra, a characteristic broad peak was observed in the region of 3000–3700 cm^−1^, with the band of highest intensity at 3316 cm^−1^, which is related to the O-H stretching vibration [30]. A broader and less-sharp peak occurs in the same wavelength range in the CS spectra, with the highest peak at 3204 cm^−1^, which also corresponds with O-H and additionally N-H stretching vibrations [31]. These bands may also be connected to the presence of water in the tested samples [32]. The bands at 2872 cm^−1^ and 2881 cm^−1^ in the CS and KGM samples, respectively, confirm the occurrence of C-H bonds that come from -CH_3_ and -CH_2_ groups being present in the carbohydrates [30].

In the KGM IR spectra, the stretching vibration coming from the C=O of the acetyl groups appears at a wavelength of 1738 cm^−1^ [33].

The peak at 1541 cm^−1^ that occurs only in the CS spectra is attributed to N-H bending vibrations [34]. The bands at around 1651 cm^−1^ and 1316 cm^−1^ confirm the presence of N-acetyl groups, represented by the C=O stretching of amide I and the C-N stretching of amide III, respectively [35]. The spectral region of 1200–900 cm^−1^ is associated with the stretching vibrations of C-O-C, C-C and C-O in the polymers’ skeletons [36]. The bands in the range from 1150 cm^−1^ to 896 cm^−1^ indicate the presence of glycosidic bonds in both of the polymers’ samples [37,38,39]. The peaks at 873 and 805 cm^−1^ are connected to the characteristic absorption bands of mannose [38,40].

FTIR spectroscopy is a very useful tool to evaluate the interactions at the molecular level between chemical groups. Intermolecular interactions may indicate the compatibility of different polymer blends. The bands’ shifts in the infrared spectrum can indicate good miscibility [40].

**Figure 2:**
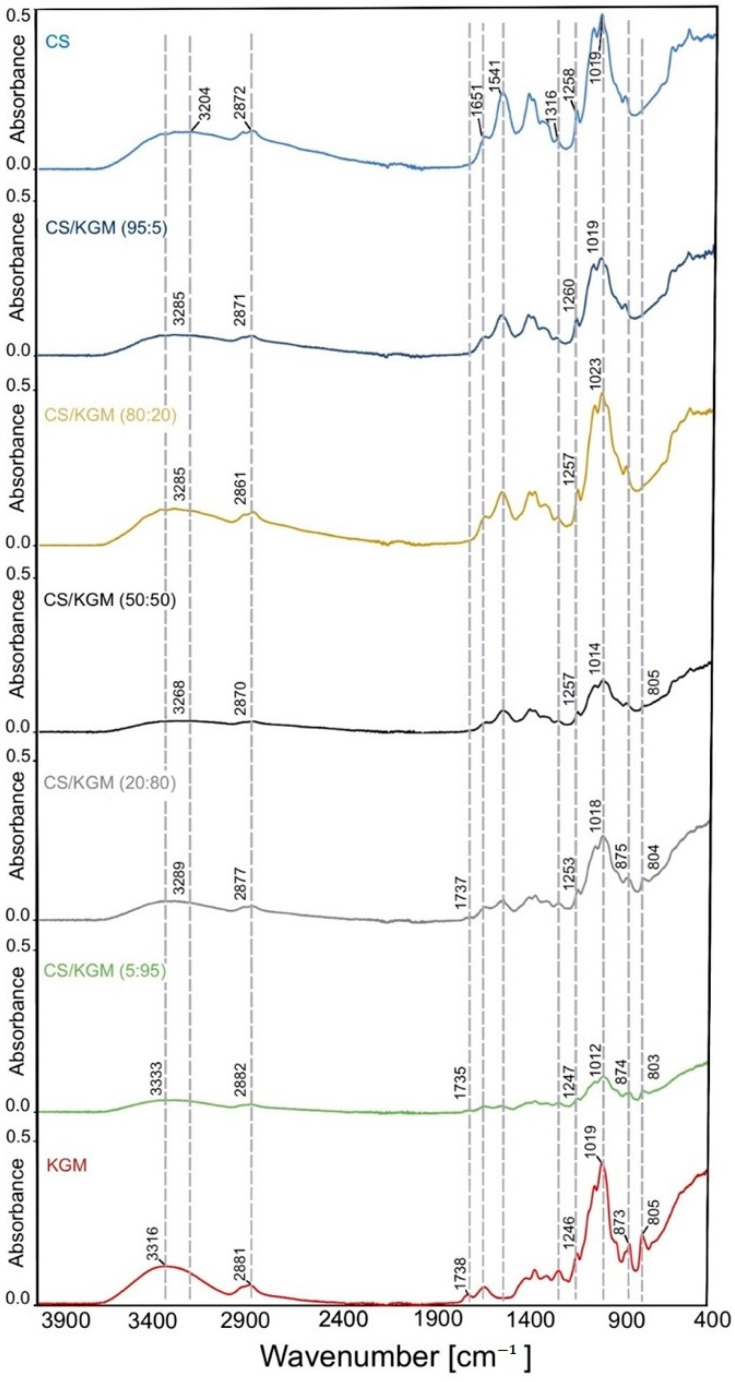
Infrared spectra of chitosan (CS), konjac glucomannan (KGM), and blended films presented in stack.

The blend films indicate shifts in the 3000–3700 cm^−1^ region that differ from the initial polymers. The wavelength values are increased in comparison with chitosan and decreased in comparison with glucomannan, ranging in between them. Similar observations apply to the C-H absorption band area: there are small shifts in the CS/KGM blend samples. The characteristic band for KGM, at about 1738 cm^−1^, disappears in the spectra of the blends with a higher content of chitosan; the last blend film with this band is CS/KGM 20:80. Even a small amount of chitosan in the blend sample causes the appearance of the band at about 1540 cm^−1^, which does not occur in konjac glucomannan. Small changes, including shifts to the lower or higher wavenumbers in the blend films’ spectra, are also observed in the 1300–1000 cm^−1^ region. The changes in the FTIR spectra also include the intensity of the bands. The highest intensity is observed in the pure chitosan and the CS/KGM 80:20 samples, and the lowest is observed in the CS/KGM 5:95 film. In light of the FTIR results, which encompass alterations in band intensity and shifts in regions, it can be posited that novel intermolecular hydrogen bonds were established. The principal functional groups engaged in this process were the hydroxyl, amine, and acetyl groups. The FTIR analysis results demonstrate that chitosan and konjac glucomannan exhibit good miscibility. The FTIR spectra for the chitosan, konjac glucomannan, and the blend films are presented in Figure 2.

#### FTIR After 6 Months of Storage

**Figure 3:**
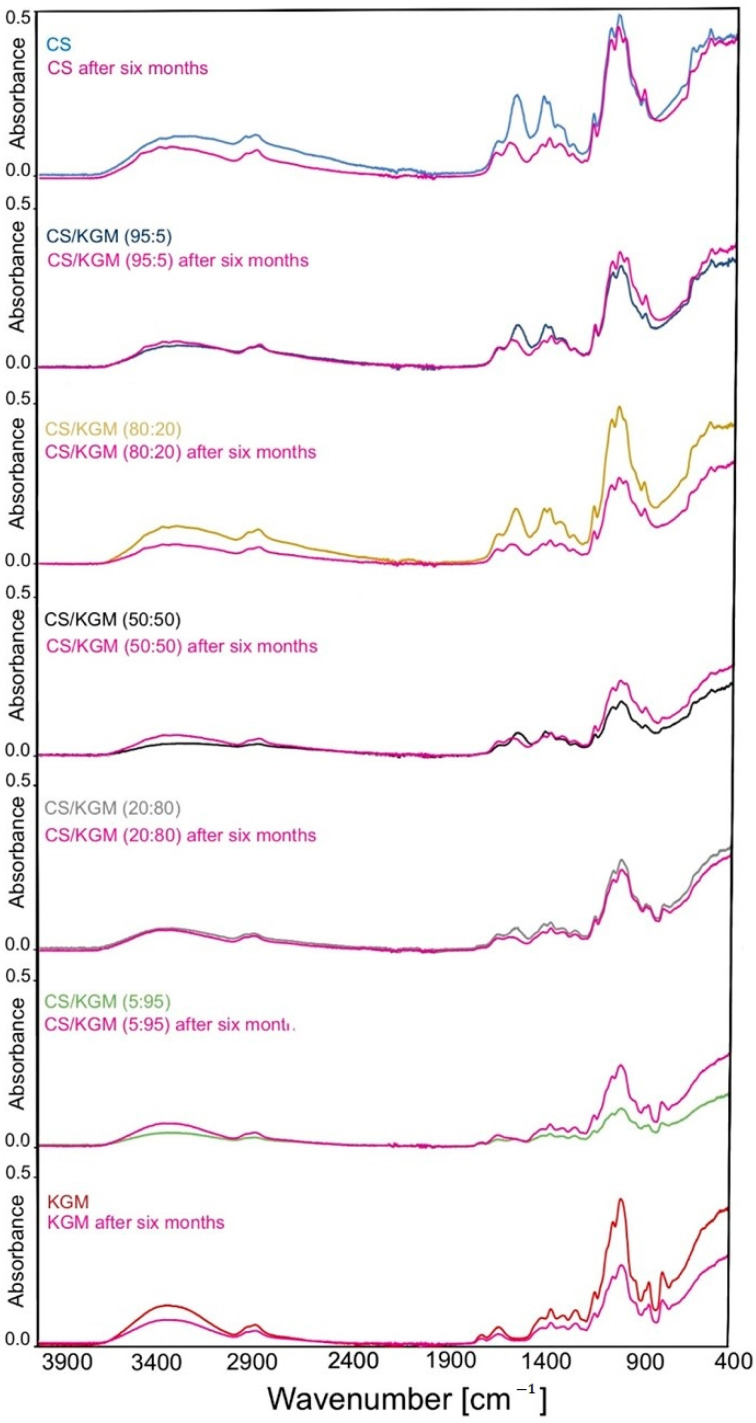
Infrared spectra of chitosan (CS), konjac glucomannan (KGM), and blended films after six months of storage, presented in stack.

The properties of the obtained blend films were also examined over time. Significant changes were observed for the pure CS film after the 6-month storage period (Figure 3). The broad band in the 3000–3700 cm^−1^ range shifted to higher wavenumbers in comparison to the initial sample (CS). This may indicate the reorganization of the hydrogen bonds in the material. Further changes include a significant reduction in the intensity of the bands at 1541 cm^−1^ and 1404 cm^−1^, as well as shifts to the higher values of wavenumber. All these changes may be caused by the water loss and chitosan chain reorganization. The pure KGM sample did not demonstrate any modifications in the appearance and localization of its bands, and only a slight increase of intensity was observed after 6 months of storage.

The main changes that affected the blend samples included the decrease of individual bands’ intensity and shifts in the same region as CS. In the two samples with the highest KGM concentrations (80:20 and 5:95), additionally, an increase of intensity at about 1000 cm^−1^ was observed. In sample 5:95, the band at about 1556 cm^−1^ disappeared in comparison to the freshly obtained sample.

### 3.2. Mechanical Testing

**Figure 4:**
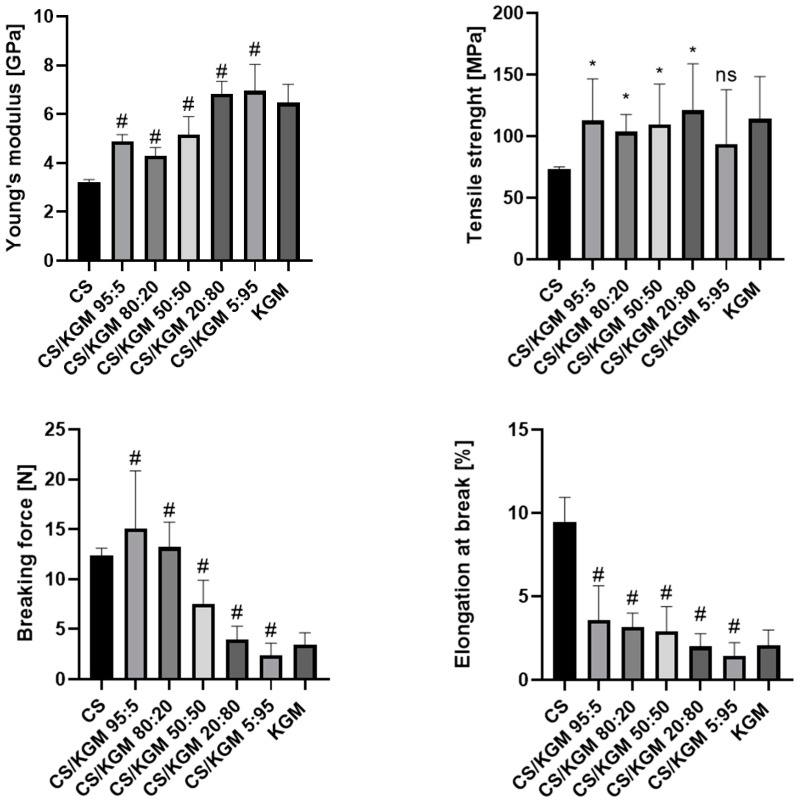
The results of mechanical parameters obtained during tensile tests for pure polymeric and blend films. Data are presented as a mean value with standard deviation. Statistically significant differences are indicated as follows: * *p* < 0.05; # *p* < 0.0001; ns—not significant.

The proportions of the blend mixtures affected the mechanical properties of the thin films significantly. The tensile test results are shown in Figure 4. The Young’s modulus value was the lowest for the pure CS samples. As the KGM content of the blends increased, higher values of this parameter were observed. This indicates the rather low deformability of the KGM films in comparison to CS.

The blend films exhibited variable values of tensile strength, the highest one belonging to CS/KGM 20:80 and the lowest to CS. The highest breaking force values were observed for the CS/KGM 95:5 samples, and the opposite observation was for CS/KGM 5:95. The decreasing trend of the parameter’s value with a higher percentage of KGM can be seen.

A similar tendency was observed for the elongation at the breaking point, with the highest value being recorded for CS and lower results being observed as the percentage of this polymer in the blends decreased.

The increased results of the Young’s modulus and the lower values of elongation at the breaking point of the blends’ films may indicate new hydrogen bond formation between the biopolymers, which may have modified their mechanical properties and increased the structural integrity of the samples.

#### Mechanical Testing After 6 Months of Storage

**Figure 5:**
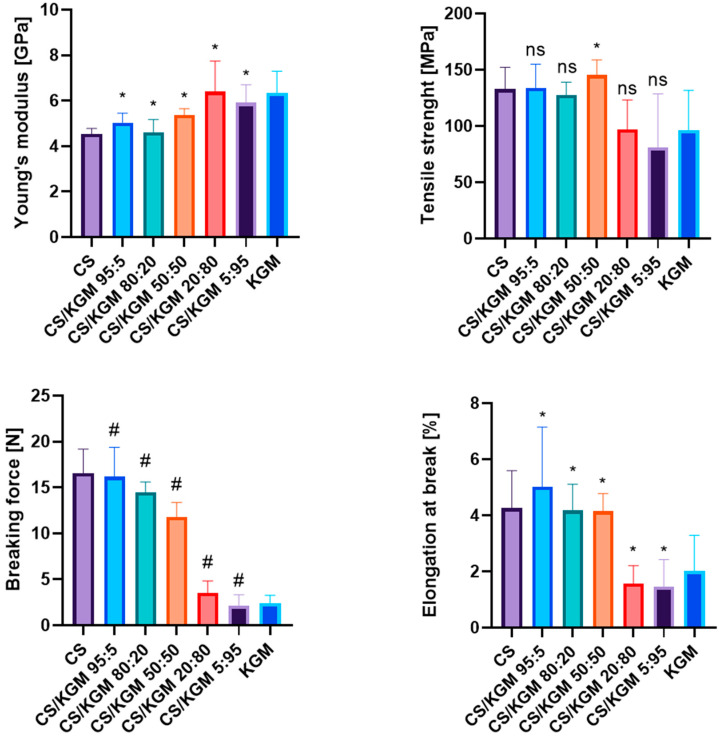
The results of mechanical parameters obtained during tensile tests for 6-month-old pure polymeric and blend films. Data are presented as mean value with standard deviation. Statistically significant differences are indicated as follows: * *p* < 0.05; # *p* < 0.0001; ns—not significant.

The six-month storage period influenced the mechanical properties of the samples (Figure 5). The most significant changes were observed for the pure chitosan film, for which the value of the Young’s modulus, tensile strength, and breaking force increased, while elongation at the breaking point decreased compared to the freshly tested sample. These observations indicate a reduction in the elasticity of the film. The passage of time had less impact on the properties of KGM, although the tensile strength decreased significantly. The blend samples also were affected by small changes in their mechanical properties. All those changes reflect the chemical modifications that occurred during storage.

### 3.3. Scanning Electron Microscopy (SEM)

**Figure 6:**
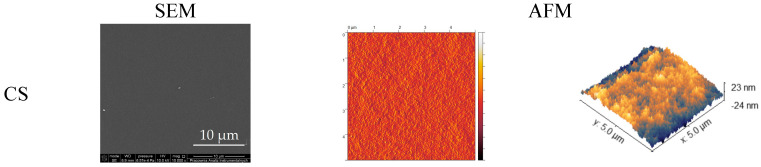

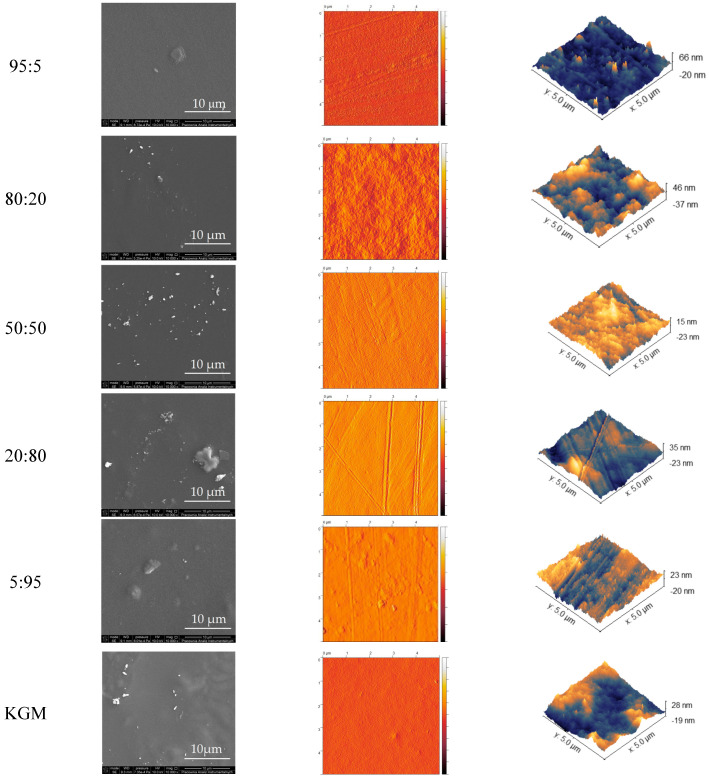
Surface structure analysis − comparison of SEM and AFM images of chitosan (CS), konjac glucomannan (KGM), and blended films.

SEM images of CS, KGM, and their blends are presented in Figure 6. The SEM analysis provided information on the surface structure of the films, which were rather uniform and smooth. The chitosan film showed the most surface uniformity. The greater the proportion of KGM in the blending ratio, the less smooth the surface was. The KGM film was characterized by a more granular structure.

### 3.4. Atomic Force Microscopy (AFM)

Figure 6 presents the atomic force microscopy (AFM) images of CS, KGM, and their blend films. The topography of the CS sample presents quite a uniform structure: the small, evenly distributed hills can be observed. The microstructure of the KGM sample is more diversified and includes changes in the level of larger areas. It corresponds with the SEM images.

The results of the tested samples’ roughness are summarized in Table 2. CS is characterized by the lowest value of roughness; the addition of KGM caused an increase in this parameter. A much higher roughness is visible in the blend proportions CS/KGM 95:5 and 80:20. The blend film with an equal amount of the two polymer solutions exhibited a similar roughness value to the initial CS sample in this parameter. The samples CS/KGM 20:80 and 5:95 had values of roughness between those of the initial polymers, but closer to the KGM. The roughness parameter helps to describe the materials’ adhesion properties. The adhesion between two materials is strongly influenced by their surfaces and contact topography. In terms of topical applications, adhesion to the skin is an extremely important parameter, which depends on the mentioned roughness, as well as the other material properties and thickness [41].

### 3.5. Thermogravimetric Analysis

**Figure 7:**
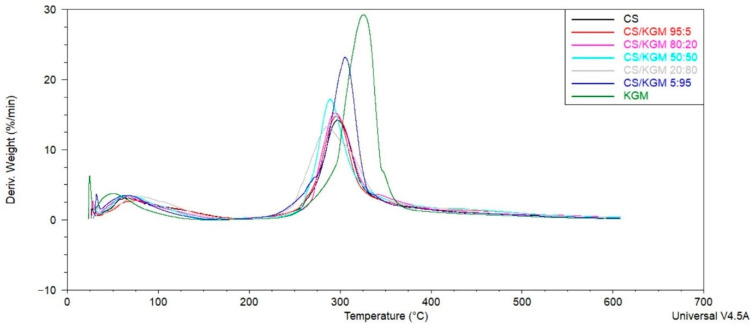

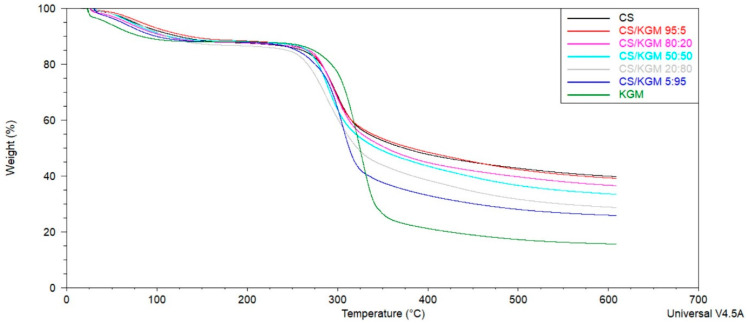
DTG and TG curves of chitosan (CS), konjac glucomannan (KGM), and the blended samples.

The thermal stability and degradation of the prepared blend films can be evaluated by the analysis of the thermal gravimetry (TG) and the differential thermal gravimetry (DTG) curves, which are shown in Figure 7. During the thermal decomposition of each film, two distinct stages were observed. The initial stage of the process entails the release of water molecules and residual solvents; in this instance, acetic acid. The initial stage takes place in a temperature range from 35 °C up to approximately 150–170 °C in all the samples. The smallest weight loss in the first stage was observed for the KGM sample (8.5%), and the highest was observed for the CS/KGM 20:80 film (12.0%); for the rest of the films, the results were close to each other, in the range of 9.5–10.6%. The secondary stage, which was identified as the most significant thermal degradation of polymers at high temperatures, exhibited a more pronounced differentiation. The initial polymers have different decomposition stabilities: KGM is more thermally stable than CS. The T_max_ value for the pure KGM was 326 °C, and additionally, the exothermic peak was highest for this biopolymer, which was connected to the highest weight loss in this step (64.7%). The pure CS T_max_ value was 297 °C. Only the sample CS/KGM 5:95 had a Tmax value higher (305 °C) than that of CS. The rest of the samples had T_max_ values closer to the CS film (Table 3). Some changes, which appeared in the DTG curves of the blend films, may suggest that a hydrogen bonding interaction was established between the chitosan and the konjac glucomannan. Importantly, in the degradation phase, only one peak can be observed in all of the curves, which may indicate good miscibility.

### 3.6. Swelling and Degradation Properties

The samples lost their weight (6.04–9.12%) in a drying process before the swelling measurements, which is connected with water and solvent residue evaporation. The results of the swelling and degradation analysis are collected in Table 4. Each result is presented as an average value with a standard deviation. All of the polymeric samples, besides the pure KGM, could swell and revealed a weight increase after being placed in the PBS solution. The KGM films were dissolved in the first 15 min of measurement. Even a small addition of CS to the KGM film (CS/KGM 5:95) caused a stability increase. The pure CS sample reached a maximum degree of swelling in one hour, after which, the values decreased, which may indicate the onset of the degradation process. Similar observations were noted for all of the samples; their weight reached a maximum at the very beginning of the measurements and then steadily decreased until the end of the experiment. KGM has a higher ability to swell than chitosan; the sample with the highest swelling degree was CS/KGM 5:95. The sample containing equal proportions of the two polymer solutions exhibited the lowest ability to swell.

#### Swelling and Degradation Properties After 6 Months of Storage

All of the samples that were measured after a 6-month period of storage were characterized by much lower swelling degrees (Table 5). These samples reached the maximum weight at a later time, and the degradation time was delayed in comparison to the films that were examined immediately after being obtained. The weight was also more stable over time. This suggests a cross-linking process during the storage period, which influences the durability and swelling ability of the materials.

### 3.7. Contact Angle and Surface Free Energy

The wettability of the CS/KGM blend films was investigated by the measurement of the materials’ contact angle using the sitting drop method. This is a very significant feature, which helps to characterize the surface of the material. It is contingent upon the physical and chemical homogeneity of the surface, in addition to the presence of surface roughness or fouling [42].

The initial films that we investigated, which were based on pure chitosan and pure konjac glucomannan, had different hydrophilic affinities (Table 6). The proportion of these two polymers in the blend films significantly affected the values of the contact angle. The CS sample was more hydrophobic, and the addition of 5% of KGM caused the increase of contact angle for glycerine. Further increasing the proportion of KGM in the sample resulted in a decrease in the wetting angle and an increase in the hydrophilic properties.

The higher the KGM proportion in the blend, the higher the share of the polar component of the surface free energy was. The highest value of this parameter was observed for the pure KGM film, which suggests that hydrogen bonds, dipole–dipole interactions, and induction forces play a significant role on the film surface [43]. Lowering the proportion of KGM in the blends resulted in a reduced occurrence of these interactions. Wettability is a crucial factor regarding many uses, including dermal applications. The enhanced hydrophilicity of the material is not conducive to optimal dermal applications, given the inherent hydrophobic character of the skin, which is largely attributable to the composition of the stratum corneum [44]. However, in the case of an injury to the skin or an open wound, the appropriate hydrophilicity facilitates a more rapid healing process. Furthermore, increased hydrophilicity is linked to an enhanced capacity to absorb wound exudate and facilitate adequate moisture within the wound environment [45].

## 4. Discussion

Carbohydrates are the most prevalent biomolecules in the natural environment and were demonstrated to confer a multitude of benefits to human health [46]. Polysaccharide-based materials are of significant value in a multitude of applications pertaining to medicine, food, and cosmetics, largely due to their biodegradability, biocompatibility, and liquid absorption properties [14,47]. In addition to their applications in the aforementioned fields, these macromolecules are also employed in the packaging industry and water purification [48,49,50,51]. These biopolymers are also environmentally friendly as they come from sustainable and renewable sources [22,52]. Chitosan and konjac glucomannan are polysaccharides that are no exception in this aspect. Chitosan- and glucomannan-based films possess specific mechanical strength and physicochemical properties that are modifiable. The blends of chitosan and glucomannan used in this study allowed the preparation of biopolymer films with different properties compared to the starting polymers.

Changes in the chemical structure of the blend films observed in this research align with observations made by other researchers. Li et al. observed that the broad absorption band at approximately 3440 cm^−1^ exhibited a shift contingent on the konjac glucomannan content of the blend. Moreover, the IR spectrum demonstrates that the band at approximately 1723 cm^−1^, which is originally present in konjac glucomannan, disappeared, and there was a shift at the wavenumber 1638 cm^−1^. The aforementioned alterations indicate augmented intermolecular hydrogen bond formation between the biopolymers [53]. Similar observations were made by Neto et al. [18]. The formation of hydrogen bonds between the -OH and -NH_2_ groups from chitosan and the -OH and -COCH_3_ groups in glucomannan, as evidenced by changes in the absorption bands, is the underlying cause of the miscibility observed between the two polymers [22].

The material’s mechanical properties are crucial for its application. For instance, products that are applied to the skin should exhibit the requisite tensile strength values (2.5 to 35 MPa) and degrees of elongation at their breaking point (70–78%), which correspond to the tensile properties of healthy human skin, which can withstand some deformations [18]. In the research conducted by Li et al., the highest value for tensile strength was observed for the sample containing 20% chitosan; a pure KGM film had a significantly higher value for this parameter than a CS film [53]. Ye et al. observed the same tendency during CS/KGM films’ mechanical examination [25]. The observations from the two studies are similar to those obtained here. A CS/KGM blend film was also examined by Shang et al.; they observed that the tensile strength of a film made using 50:50 proportions of the polymers was very low [54], which remains contrary to our observations. However, the reagents used in their study had different chemical characteristics compared to our polymers. These differences include their molecular weights and the deacetylation degree of chitosan, which may have affected the final properties of material. Fan et al. obtained the fibers in the spinning process that was based on CS/KGM blends in different proportions. The mechanical tests demonstrated that the incorporation of KGM into CS enhances the tensile strength of the fibers. Furthermore, the mechanical properties of the blend can be optimized by controlling the blend conditions [55]. Chen et al. examined the mechanical properties of the chitosan/konjac glucomannan bilayer, and their observations of the degree of elongation at the breaking point show a similar trend to our research. The pure CS film had the highest value of this parameter. The higher the KGM content in the composite, the lower the percentage of elongation at the breaking point was [56]. Pure KGM film materials tend to have poor mechanical properties, and the degree of elongation at the breaking point is one of the indicators of this condition.

The surface morphology of the CS/KGM blends’ films was examined by SEM and AFM techniques. Our observations corroborate the findings reported by Xiao et al., whereby the incorporation of KGM into CS resulted in a reduction in the samples’ homogeneity, which may suggest a decline in miscibility. However, the samples with a low content of KGM were characterized by smooth and homogenous surfaces [22]. Similar insights were presented by Zou et al. in their research on composite films made of corn starch and konjac glucomannan. The addition of KGM resulted in a more uneven and granular surface structure [33]. Nair et al. also observed small particles on the film surface and suggested that these constituted less soluble fractions of KGM [57].

The thermal stability of the tested blends and the initial biopolymers was evaluated by a thermogravimetric analysis. We demonstrated that the thermal stability of the films is directly proportional to the KGM content of the blend. Neto et al. obtained similar results. All of the blend films that they tested showed the values of the maximum weight loss temperature as being between those of the initial polymers [18]. The same findings were also presented by Xiao et al. and Xu et al. [22,40]. All of these changes suggest that new hydrogen interactions were established between CS and KGM. What is more, the DTG curves from both this research and the mentioned studies confirmed the good miscibility of CS and KGM over all of the composition ranges.

The swelling properties of materials that are designed for skin applications, such as wound adhesive, are extremely important. Two aspects must be considered: providing ideal moisture conditions and absorbing the exudate from the wound, depending on the wound type [58]. The modification of chitosan film by blending with konjac glucomannan increases the swelling ability of the material, especially when evaluated after a long storage period. The results of a water swelling properties analysis of CS/KGM films performed by Xiao et al. showed higher levels of swelling for all the blended samples than for pure chitosan [22]. What is more, a pure KGM sample was dissolved immediately after immersion in liquid, as took place in our study. Fan et al. evaluated the water-retention properties of a CS/KGM blend fiber; the lowest value was observed for pure CS [55]. In a study conducted by Hua et al., blend films composed of chitosan and konjac glucomannan carboxymethylated form were examined. A content of more than 30% of KGM derivative in a film resulted in a notable enhancement of its swelling ability [59]. Konjac glucomannan has a great water absorption capacity, which can be as much as up to 100 g of water per 1 g of konjac [60]. The high swelling degree of the films with high KGM contents that are presented in our study remains consistent with the existing theory. Sample 5:95 achieved 946%, the highest result of all the samples tested. Wu et al. presented a study of a superabsorbent polymer based on KGM. Their product reached almost the maximum swelling value in the first hour of analysis, which is in line with our research [61]. The hydrophilic character of this polysaccharide is the consequence of the hydroxyl group’s occurrence in the backbone, which promotes water uptake by the biomaterial. More recently, Chen et al. prepared bilayers of chitosan and konjac glucomannan in different weight ratios. The sample with an equal ratio of CS and KGM exhibited the lowest swelling properties [56]. Although the system tested was a composite, the observations on it were similar to our blend sample of CS/KGM 50:50. One explanation for this phenomenon may be the high proportion of hydrogen bonds formed between the two polymers, which limits hydrophilic interactions with water molecules [56]. The results of the swelling analysis permitted the assessment of the onset of degradation processes, which commenced, for all of the samples, approximately one hour after the start of the analysis. In a study conducted by Tkongachai et al., a film composed of chitosan and collagen was examined. The greatest degradation level was observed at the beginning of the analysis. Following the third day, the percentage of degradation was observed to be lower and was maintained at a comparable point [62]. Over the course of our study, the degradation process started within the first hours of the test period. Furthermore, after the third day, the reduction in the samples’ weight was less pronounced and exhibited greater stability over time.

The contact angle measurements confirmed the hydrophilic character of all of the tested films. The lowest values of the contact angle were observed for pure KGM. The higher the KGM content in the blend samples, the more hydrophilic the character of the film was. Similar insights were observed by Strnad et al.; they examined keratin films enriched with konjac glucomannan. Although the keratin film had hydrophobic properties, the addition of KGM caused the change of this state toward hydrophilic characteristics [63]. Qin et al. performed an investigation on the drying temperature’s influence on konjac glucomannan/agar blend films. The measurements of the contact angle for pure KGM were the lowest, which remains consistent with the previous observations. Controlling the drying temperature enabled the modification of the water-spreading properties [64].

The results obtained from the materials tested after a specified period of storage demonstrate that the time since the material was obtained has a discernible impact on its properties. These changes include a reduction in their mechanical properties, as evidenced by a loss of flexibility in the samples. The swelling results also indicate a reduction in fluid absorption, which has implications for the functional characteristics of such materials. Depending on the application, these changes can be both positive and negative. In the case of materials such as wound dressings, where the fit to the skin and the ability to absorb fluids is important, the biopolymer materials from this research would not perform as well after prolonged storage.

Biopolymers constitute a diverse class of macromolecules with potential applications in the cosmetic industry. The group includes polysaccharides, which are of particular interest to the cosmetic industry, primarily due to their safety profile. The application of these compounds does not carry the risk of zoonotic diseases, which is a concern in the case of collagen [65]. Polysaccharides were shown to have a range of beneficial effects on the skin, including moisturizing, antioxidant, and anti-ageing properties [66]. In addition to their safety and efficacy, the naturality and eco-friendliness of these compounds are becoming increasingly important considerations for consumers, producers, and researchers [67,68]. Chitosan, due to its polycationic nature, is capable of interacting with the skin and damaged hair [69]. Additionally, konjac glucomannan has been shown to possess a regenerative potential for the skin [15]. Both biopolymers possess film-forming properties. This characteristic is highly beneficial in the development of cosmetic products. Polymeric films can be simply and effectively applied to the skin, for example as a beauty mask or an eye patch. [15,68,70,71,72].

The same properties that are advantageous in cosmetic applications are of paramount importance in the development of solutions for medical and tissue engineering purposes, for instance, in wound dressings [19,73,74,75,76,77,78]. Wound adhesives are the first line in wound management; however, traditional medical materials, such as bandages or gauze, have some disadvantages, like poor springiness. Materials based on biopolymers overcome these limitations [79]. Biopolymers, including those used in this study, have a caring or regenerative effect themselves in the aforementioned areas. However, they are frequently employed as a matrix for the incorporation of active substances or drugs. Zeng et al. developed a hydrogel intended for wound healing, consisting of konjac glucomannan and xanthan gum, containing polydopamine nanoparticles. The results of the in vivo study demonstrated the efficacy of the hydrogel formulations in wound treatment, both with and without nanoparticles [79]. Another example is a study conducted by Jiang et al., in which a chitosan/konjac glucomannan matrix incorporated silver nanoparticles [80]. The studies using chitosan and konjac glucomannan as matrices for active compounds for wound healing are much more prevalent, including investigations of the addition of berberine [81], exosome nanoparticles [82], paeoniflorin and zinc nanoparticles [83], gentamicin [84], stevioside-stabilized honokiol [85], or graphene oxide [86]. In the context of the biomedical and cosmetic applications of biopolymers, the role of hydrogels is a fundamental aspect that cannot be overlooked. The -NH_2_ and -OH groups present in chitosan and glucomannan, respectively, allow for modifications to be made, including the formation of spatial networks and the creation of hydrogels [87]. Hydrogels possess the remarkable ability to swell and can also act as a reservoir for an active substance, which is the reason for their extensive utilization in drug delivery systems [88] and wound dressings [21]. The blending of two polymers represents the most straightforward physical method of modifying a polymer matrix in this manner [87]. Konjac glucomannan displays a particular potential for use as a hydrogel, given its high swelling capacity. However, its standalone application is constrained by its poor mechanical properties in solutions [21]. The combination with another polymer, in this case chitosan, markedly enhances this parameter. This offers the possibility of further investigation into the potential of the chitosan/konjac glucomannan combination, for example through the utilization of alternative, more complex cross-linking techniques.

The materials presented in this study are in line with modern requirements for cosmetics, wound dressing, or food packaging. Nevertheless, this is merely a preliminary investigation that requires further expansion in the future. However, it represents an excellent point of departure. Although there are already some studies on chitosan and konjac glucomannan in the areas outlined, their blends in the proportions proposed here have not yet been investigated.

## 5. Conclusions

We successfully obtained blend films based on two biodegradable and biocompatible polysaccharides—chitosan and konjac glucomannan. The implemented method of film production was simple and low-cost. The test methods used in this study made it possible to assess the miscibility of the biopolymers, which was good in all of the composition ranges. The infrared spectra analysis confirmed the chemical structure of pure polymers and enabled the evaluation of any changes found in blend films. It can be posited that the formation of hydrogen bonds was facilitated by the involvement of the -OH, -COO-, -NH, and COCH_3_ groups. The mechanical properties of the blend samples were modified in comparison to the initial polymers’ films. Generally, the addition of KGM to CS increased the structural integrity of the samples. The highest tensile strength value was exhibited by the CS/KGM 20:80 sample. SEM and AFM images showed that the higher the KGM content in the blend composition, the less the homogeneity of the film’s surface. The highest roughness was shown by the CS/KGM 80:20 sample. The KGM content in the biopolymeric films increased their thermal stability. The swelling ability of the tested samples was significantly increased by higher KGM concentrations within the blends. The contact angle result revealed the high impact of the KGM content on the hydrophilicity of the samples. In this research, we also evaluated the effects of the passage of time on some of the films’ parameters. Such an assessment is extremely important from the point of view of product storage. The infrared spectra showed changes in band intensity and some shifts compared to the previously examined samples. These changes could be connected to water loss, as well as the cross-linking processes. The results of the mechanical parameters assessment and the result of the swelling degree analysis reflect the changes that occurred in the chemical composition of the films.

We can conclude that films based on a combination of chitosan and konjac glucomannan are promising for use as matrices for many applications. The shape of the film predisposes the material to surface and packaging applications. Some aspects of the first-mentioned usage have been taken into account when investigating the properties of the obtained materials. In particular, the swelling and AFM results suggest that the addition of KGM to the samples improves their application properties. Some of the blending ratios proposed in this study and the testing of the properties of the samples after a certain storage period are new approaches. This study contributes to the current research on biomaterials for surface applications, specifically on materials based on the chitosan/konjac glucomannan combination. These biopolymer blend materials can be further modified; this is a prelude to further work in this area.

## Figures and Tables

**Table 1:** The overview of chitosan (CS), konjac glucomannan (KGM), and their blends’ film appearance.

CS	CS/KGM, 95:5	CS/KGM, 80:20	CS/KGM 50:50	CS/KGM 20:80	CS/KGM 5:95	KGM
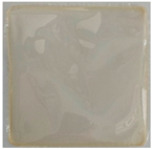	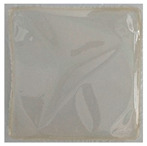	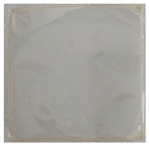	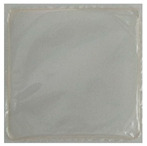	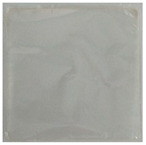	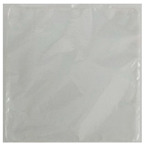	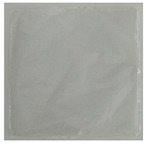
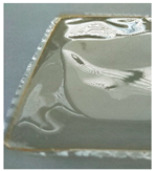	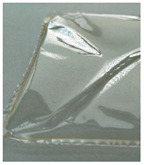	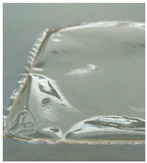	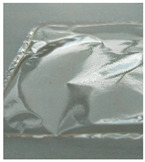	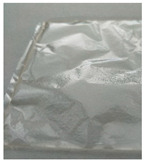	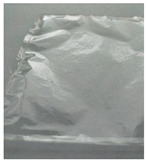	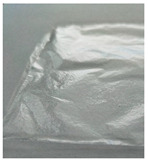

**Table 2:** The roughness values of chitosan (CS), konjac glucomannan (KGM), and blended films.

Sample	R_q_ [nm]	R_a_ [nm]
CS	5.64 ± 1.25	4.50 ± 0.97
CS/KGM 95:5	9.60 ± 2.85	6.35 ± 1.04
CS/KGM 80:20	12.60 ± 1.14	9.88 ± 0.77
CS/KGM 50:50	5.95 ± 2.48	4.69 ± 1.92
CS/KGM 20:80	6.57 ± 0.84	4.96 ± 0.46
CS/KGM 5:95	6.17 ± 0.75	4.86 ± 0.50
KGM	6.87 ± 0.94	5.68 ± 0.98

**Table 3:** Summary of temperature maximum values for decomposition stages for all tested samples.

Sample	T_max1_ [°C]	T_max2_ [°C]
CS	62.87	296.72
CS/KGM 95:5	74.31	296.01
CS/KGM 80:20	65.02	293.86
CS/KGM 50:50	67.16	288.86
CS/KGM 20:80	75.74	285.99
CS/KGM 5:95	65.73	305.30
KGM	50.00	326.04

T_max_—represents the maximum temperature value for a given stage.

**Table 4:** The results of the swelling and degradation analysis for the chitosan (CS), konjac glucomannan (KGM), and blended films, obtained over two weeks (presented as a mean value of weight change [%] with a standard deviation).

Specimen	0.25 h [%]	1 h [%]	2 h [%]	4 h [%]	8 h [%]	24 h [%]	48 h [%]	72 h [%]	168 h [%]	336 h [%]
CS	270 ± 23	279 ± 36	235 ± 16	207 ± 11	189 ± 10	184 ± 5.0	166 ± 9.0	148 ± 5.0	137 ± 4.0	128 ± 7.0
CS/KGM 95:5	236 ± 82	241 ± 100	198 ± 15	173 ± 10	159 ± 12	141 ± 11	134 ± 9.0	124 ± 7.0	119 ± 20	107 ± 20
CS/KGM 80:20	296 ± 44	269 ± 33	230 ± 17	218 ± 10	189 ± 16	177 ± 8.0	152 ± 13	149 ± 9.0	141 ± 3.0	124 ± 8.0
CS/KGM 50:50	162 ± 21	159 ± 27	149 ± 24	144 ± 8.0	144 ± 10	138 ± 9.0	125 ± 15	123 ± 5.0	120 ± 10	122 ± 12
CS/KGM 20:80	310 ± 184	265 ± 117	256 ± 112	238 ± 97	242 ± 91	255 ± 107	247 ± 108	242 ± 109	233 ± 114	229 ± 101
CS/KGM 5:95	946 ± 178	938 ± 95	886 ± 45	841 ± 54	825 ± 52	765 ± 41	761 ± 35	670 ± 51	636 ± 60	587 ± 24
KGM	-	-	-	-	-	-	-	-	-	-

**Table 5:** The results of the swelling and degradation analysis for the chitosan (CS), konjac glucomannan (KGM), and blended films (after six months of storage), obtained over two weeks (presented as a mean value of weight change [%] with a standard deviation).

Specimen	0.25 h [%]	1 h [%]	2 h [%]	4 h [%]	8 h [%]	24 h [%]	48 h [%]	72 h [%]	168 h [%]	336 h [%]
CS	54 ± 12	76 ± 20	78 ± 36	78 ± 17	69 ± 13	69 ± 15	72 ± 26	61 ± 15	61 ± 19	62 ± 25
CS/KGM 95:5	87 ± 16	87 ± 3.0	87 ± 4.0	83 ± 3.0	82 ± 3.0	77 ± 4.0	81 ± 1.0	73 ± 4.0	67 ± 2.0	70 ± 5.0
CS/KGM 80:20	85 ± 4.0	87 ± 5.0	87 ± 1.0	82 ± 3.0	84 ± 5.0	84 ± 5.0	78 ± 4.0	76 ± 4.0	77 ± 1.0	73 ± 3.0
CS/KGM 50:50	99 ± 3.0	89 ± 4.0	99 ± 10	105 ± 8.0	102 ± 7.0	83 ± 2.0	88 ± 5.0	96 ± 7.0	86 ± 5.0	90 ± 4.0
CS/KGM 20:80	187 ± 74	168 ± 30	149 ± 14	135 ± 12	137 ± 16	130 ± 3.0	125 ± 5.0	126 ± 8.0	116 ± 6.0	126 ± 8.0
CS/KGM 5:95	373 ± 22	413 ± 45	431 ± 21	430 ± 22	413 ± 26	398 ± 18	410 ± 18	387 ± 23	379 ± 19	367 ± 17
KGM	-	-	-	-	-	-	-	-	-	-

**Table 6:** The values of the contact angle and surface free energy for chitosan (CS), konjac glucomannan (KGM), and blended films.

Specimen	Θ^G^	Θ^D^	γ_s_ [mJ/m^2^]	γ_s_^d^ [mJ/m^2^]	γ_s_^p^ [mJ/m^2^]
CS	90.08 ± 2.93	47.33 ± 9.46	35.50 ± 5.13	35.23 ± 5.41	0.40 ± 0.1
CS/KGM 95:5	97.82 ± 3.00	64.52 ± 2.48	25.97 ± 1.14	25.62 ± 0.89	0.35 ± 0.25
CS/KGM 80:20	89.33 ± 2.87	52.07 ± 6.40	32.73 ± 3.59	31.94 ± 3.56	0.79 ± 0.04
CS/KGM 50:50	81.83 ± 3.53	29.54 ± 7.94	43.88 ± 2.99	43.12 ± 2.73	0.77 ± 0.26
CS/KGM 20:80	75.19 ± 6.94	39.57 ± 8.63	39.30 ± 4.54	35.86 ± 3.14	3.43 ± 1.40
CS/KGM 5:95	76.11 ± 2.57	43.94 ± 3.34	37.32 ± 1.94	33.76 ± 1.51	3.55 ± 0.45
KGM	56.27 ± 3.74	35.24 ± 1.30	45.35 ± 1.69	35.58 ± 0.20	11.77 ± 1.89

## Data Availability

The original contributions presented in the study are included in the article; further inquiries can be directed to the corresponding author.
